# Top-Down Computerized Cognitive Remediation in Schizophrenia: A Case Study of an Individual with Impairment in Verbal Fluency

**DOI:** 10.1155/2015/242364

**Published:** 2015-04-09

**Authors:** Marjolaine Masson, Til Wykes, Michel Maziade, Clare Reeder, Marie-Anne Gariépy, Marc-André Roy, Hans Ivers, Caroline Cellard

**Affiliations:** ^1^École de Psychologie, Université Laval, Québec, QC, Canada G1V 0A6; ^2^Institute of Psychiatry, King's College London, London SE5 8AF, UK; ^3^Centre de Recherche de l'Institut Universitaire en Santé Mentale de Québec, Québec, QC, Canada G1J 2G3; ^4^Département de Psychiatrie, Faculté de Médecine, Université Laval, Québec, QC, Canada G1V 0A6

## Abstract

The objective of this case study was to assess the specific effect of cognitive remediation for schizophrenia on the pattern of cognitive impairments. Case A is a 33-year-old man with a schizophrenia diagnosis and impairments in visual memory, inhibition, problem solving, and verbal fluency. He was provided with a therapist delivered cognitive remediation program involving practice and strategy which was designed to train attention, memory, executive functioning, visual-perceptual processing, and metacognitive skills. Neuropsychological and clinical assessments were administered at baseline and after three months of treatment. At posttest assessment, Case A had improved significantly on targeted (visual memory and problem solving) and nontargeted (verbal fluency) cognitive processes. The results of the current case study suggest that (1) it is possible to improve specific cognitive processes with targeted exercises, as seen by the improvement in visual memory due to training exercises targeting this cognitive domain; (2) cognitive remediation can produce improvements in cognitive processes not targeted during remediation since verbal fluency was improved while there was no training exercise on this specific cognitive process; and (3) including learning strategies in cognitive remediation increases the value of the approach and enhances participant improvement, possibly because strategies using verbalization can lead to improvement in verbal fluency even if it was not practiced.

## 1. Introduction

Cognitive impairments are a core feature of schizophrenia with impairments in nearly every cognitive domain [1, 2]. A recent meta-analysis [2] demonstrated that patients with schizophrenia scored significantly lower than did controls across all cognitive tests and domains with largest impairments in processing speed and episodic memory [2, 3]. While 70%–80% of individuals with schizophrenia demonstrate cognitive impairments, one standard deviation below the mean of healthy comparison subjects (i.e., 16th percentile) [3, 4], relative to the general population, nearly 100% demonstrate decreased performance relative to their own premorbid cognitive status [5, 6]. Cognitive impairments in attention, verbal memory, and executive functioning have demonstrable prognostic value; that is, the degree of impairment predicts ability to achieve functional goals through treatment [7–9].

In considering the improvement of functioning, it is important to take account of any specific cognitive difficulties that are related to functioning outcomes. Episodic memory is one of the cognitive domains usually associated with poor social functioning [10]. However, one overlooked cognitive deficit that can negatively impact social life is verbal fluency [10]. Verbal fluency is generally considered as a measure of executive functions. To assess verbal fluency, participants are asked to name as many items as possible from a given category in a given time period. The category may be semantic, such as animals or types of fruit, or phonemic, such as words that begin with the letter *p* [11]. Some studies have demonstrated that patients with schizophrenia produce fewer items and do not use recall strategies (e.g., farm animals and wild animals) during letter and semantic fluency tests [12]. Impairment in verbal fluency was more severe for semantic fluency (effect size = 1.21) than for letter fluency (effect size = 0.98) [2, 13].

Considering the generalized cognitive impairments, several psychological treatment strategies have therefore been developed to improve cognitive functioning in this population and they are referred to as cognitive remediation strategies. As defined at the Cognitive Remediation Experts Workshop (Florence, Italy, April 2010), cognitive remediation for schizophrenia refers to “a behavioral training based intervention that aims to improve cognitive processes (…) with the goal of durability and generalization.” Two recent meta-analyses [14, 15] demonstrated that cognitive remediation for individuals with schizophrenia has a positive, moderate effect on overall cognition, psychosocial functioning, and symptoms. Moreover, greater effects on psychosocial functioning were observed when cognitive remediation was combined with psychiatric rehabilitation [14]. However, transfer of gains from the research context to everyday life is of moderate effect size across cognitive rehabilitation studies that included measures of psychosocial functioning [14, 15]. Cognitive remediation tasks are typically quite structured and are therefore significantly different from the situations that individuals with schizophrenia encounter in everyday life [16]. Currently, cognitive remediation with schizophrenia patients includes two primary approaches: “drill and practice” and “drill, practice, and strategy” [14]. The first is a bottom-up approach that trains cognitive processes by repetition, using exercises that focus on specific impaired processes [17]. The second is a top-down approach that also trains cognitive processes by repetition. However, this approach also provides patients with strategies for applying the practiced processes in daily life [18, 19]. That is, the “drill, practice, and strategy” remediation approach provides cues for managing real-life situations. Cognitive remediation programs that incorporate strategies and methods for addressing beliefs and motivation, rather than relying solely on drill and practice, are associated with more positive psychosocial outcomes [20]. Moreover, in developing interventions for improving social functioning in this population, it has been useful to conceptualize communication behavior in terms of social skills and constituent elements of social skills [21]. One such cognitive remediation program for individuals with schizophrenia is the Computerized Interactive Remediation of Cognition Training for Schizophrenia, CIRCuiTS [22]. The CIRCuiTS program targets difficulties with goal-directed behavior in daily life. The objectives of the program are to develop a list of individualized goals, to create an action plan for cognitive training and to implement strategies designed to facilitate the transfer of skills into everyday life.

Patients with schizophrenia have heterogeneous cognitive profiles. The CIRCuiTS program software addresses multiple cognitive processes and allows therapists to remove or adjust exercises to adapt therapy sessions as needed. To highlight specific cognitive deficits that are less visible in a larger sample, the present paper presents a case study of cognitive remediation in a patient with schizophrenia. The patient (Case A) demonstrated global cognitive impairment, with specific impairment in verbal fluency. He participated in a three-month general top-down cognitive remediation program. We hypothesized that Case A would improve performance in the cognitive domains observed to be impaired at neuropsychological baseline and targeted by the CIRCuiTS program. Moreover, as verbalization is one of the primary strategies in the CIRCuiTS program, we anticipated that this may have a beneficial effect on verbal fluency even though it is not directly targeted by specific exercises.

## 2. Case Report

This research project was approved by the appropriate ethics committee at* Centre de Recherche de l'Institut Universitaire en Santé Mentale* in Quebec City, Canada. Participants with schizophrenia were recruited and consented to participate. They were informed that they could withdraw participation at any time. The current paper presents a case study drawn from the larger sample recruited for this project. Case A was a young participant with recent-onset schizophrenia.

The inclusion criteria for the larger study were (1) confirmed* DSM-IV-TR* [23] schizophrenia diagnosis within the last ten years; (2) clinical status permitting reliable cognitive assessment; and (3) cognitive difficulties in visual episodic memory, immediate recall or delayed recall below the 16th percentile, as measured by the Rey complex figure test (RCFT). Exclusion criteria were (1) brain and metabolic disorders known to cause neuropsychological impairments; (2) substance dependence within the past six months; and (3) Intelligent Quotient below 70.

Case A was a 33-year-old man who lived alone. He had completed 13 years of education and had obtained a professional certificate in institutional plumbing. He was diagnosed with depression at age 28 and with schizophrenia (*DSM-IV*) at age 30. He had a past history of amphetamine dependence. His pharmacological treatment, which did not change throughout the intervention, included metformin (2 × 850 mg) for diabetes, modafinil (100 mg) to decrease sleepiness, atorvastatin (10 mg) for hypercholesterolemia, atenolol (12 mg) for recurrent migraines, clozapine (200 mg h.s.), desvenlafaxine (50 mg) for musical obsessions meeting criteria for obsessional compulsive disorder (in complete remission throughout the intervention), and two drops of atropine (1% h.s.) for hypersialorrhea. The patient met with his psychiatrist approximately once per month and with his psychologist and occupational therapist twice per month. Socially, Case A saw only members of his family once or twice a week; he has worked as a plumber until 2008 and has been unemployed since 2008.

### 2.1. Cognitive Complaints

At the first therapy session, Case A's therapist presented the cognitive remediation program and explained the study procedure in detail. Difficulties in daily life were also discussed. Case A reported the following cognitive complaints: difficulty retaining information, concentration and comprehension problems, and memory loss. The set of goals of cognitive remediation treatment was jointly agreed by both therapist and patient. Case A's objectives derived directly from his complaints and included the following: read a newspaper article and understand the text, read an entire book and retain the thread of the plot, and watch a movie without the use of the “pause” or “rewind” buttons.

## 3. Materials and Methods

### 3.1. Neuropsychological and Clinical Assessments

Nine neuropsychological tests and two clinical tests were used to assess Case A before the cognitive remediation (Table 1). When a score below the 16th percentile was observed in at least one variable of a cognitive domain, then the cognitive domain was considered as impaired.

### 3.2. Procedure

The experimental design included three steps: baseline assessment, a three-month cognitive remediation (CIRCuiTS), and posttest assessment. Baseline and posttest assessments both consisted of neuropsychological and clinical assessments; neuropsychological assessment was conducted by a research assistant, and clinical assessment was conducted by Case A's treating psychiatrist who had treated him since the beginning of treatment.

#### 3.2.1. Baseline Assessment

At baseline, Case A demonstrated pathological scores (<5th percentile) or impairments/difficulties (<16th percentile) in four of the nine cognitive domains assessed with neuropsychological tests (Table 2): visual episodic memory (immediate and delayed recalls), selective attention (inhibition variable), initiation/strategic search (phonemic and semantic categories), and problem solving (high trial number of first category in a card sorting test). Poor verbal fluency was also qualitatively observed during the baseline evaluation session (i.e., poverty of speech). Case A's scores were within the normal range (from 21st to 97th percentiles) for all of the remaining cognitive processes assessed (verbal episodic memory, working memory, sustained attention, and planning). Finally, Case A seemed to have good metacognitive skills because his cognitive deficits were congruent with his cognitive complaints (retaining information, concentration and comprehension problems, and memory loss).

#### 3.2.2. Cognitive Remediation

The cognitive remediation program used is named CIRCuiTS [22]. Several cognitive remediation programs exist. The originality of CIRCuiTS can be summarized in key points. First, CIRCuiTS is theory-driven based on a metacognitive model of the relationship between cognitive and functional change. Using a strategy-based approach, CIRCuiTS has an integrated focus on the transfer of cognitive skills to daily activities. It aims to develop metacognitive regulation and metacognitive knowledge, which are hypothesized to be important for the appropriate generalization of cognitive skills to daily living [24]. The focus on transfer also comes from its use of real-world goals, homework to facilitate in vivo use of new strategies, and a formulation-based approach in which the impact of cognitive strengths and difficulties on daily living skills is considered. All of these factors are known to be associated with increased motivation [25, 26]. Second, CIRCuiTS has high feasibility and acceptability among both service users and therapists as seen in a series of quantitative and qualitative studies designed to inform and test the development of CIRCuiTS [27]. Third, CIRCuiTS has high adaptability to individual differences because it is based on a flexible modular system [28].

CIRCuiTS is a computerized psychological therapy program; although patients can use the program independently, it is ideally administered by a therapist. Various cognitive training techniques and strategies are employed, including simplification and errorless learning. Those techniques have been proven to be effective in improving cognitive performance in empirical studies [24]. CIRCuiTS was designed to be completed in forty therapy sessions, at a minimum of three sessions per week. Therapy sessions last up to one hour, but therapists can adjust time according to the patient.

A therapy session consists of multiple (approximately 4–8) activities covering a wide range of cognitive functions: attention, memory, executive functions, visual-perceptual processing, and metacognitive skills. The activities are designed to target verbal skills, nonverbal skills, or both. Tasks are rotated to be diverse, interesting, and engaging for participants. The CIRCuiTS program includes two types of tasks: (1) abstract tasks, which are designed to improve cognitive functions in an abstract context (e.g., remembering a list of words), and (2) exercises, that is, complex tasks designed to reflect everyday activities (e.g., make a daily schedule and read a letter). Whereas abstract tasks are performed throughout the CIRCuiTS program, exercises are introduced gradually across sessions; the final sessions of the program consist primarily of exercises. The rationale is that patients learn new cognitive skills in an abstract context and subsequently gradually transfer the skills to everyday life. That overall objective of the program is for participants to eventually apply cognitive skills and new strategies developed in therapy in daily life.

### 3.3. Therapist Strategies

Verbalization of cues, prompts, and strategies for completing a given task are key therapist strategies. During a task, the therapist verbalizes hints for the participant, in order to facilitate mentalization of relevant strategies. Verbalized prompts are often used repetitively; participants becomes increasingly independent as therapy progresses, with the therapist verbalising key instructions at first and the participant gradually taking over the verbalisation process which occurs first overtly (out loud) and, later, covertly (mentally). For example, in the “learning a list” task, the therapist may encourage the participant to repeat the list of words to him or herself.

### 3.4. Clinical Hypotheses

Given a program that specifically addressed and targeted many of Case A's particular deficits, we expected improvement in each of his impaired scores. Improvements were expected in visual episodic memory (as measured by the RCFT), selective attention/inhibition (as measured by the Stroop inhibition test), and problem solving (as measured by the WCST). The underlying skills of verbal fluency were not directly practiced in CIRCuiTS. However, verbalization is one of the primary strategies used by the therapist and the patient during the therapy. Improvement in verbal fluency was therefore expected. No baseline deficits were observed in verbal episodic memory, working memory, or sustained attention and planning, and therefore no significant improvements were expected.

### 3.5. Statistical Analysis

Two approaches were adopted to estimate changes on main outcomes after cognitive remediation. First, the Reliable Change Index (RCI) [29] was calculated to determine whether posttherapy change observed for each variable for each participant reaches significance level. RCI, similar to a Z-change score, (a) allows estimating the extent by which a patient distance themselves from a distribution of similar symptomatic patients who were not exposed to the intervention, while (b) controlling for the instrument reliability and (c) allowing a conclusion about the “statistical significance” of the change. RCI larger than 1.96 are seen as statistically significant at a two-tailed 5% alpha level. It can be noted that this statistical approach is quite conservative [30]; a minimum change of two standard deviations is required to be considered significant. The conservative threshold means that an observed change is unlikely to be attributable to simple measurement unreliability or practice effect within pre- and posttests [31].

The RCI were computed with these variables. The standard deviations for the neuropsychological variables were gathered from our laboratory (M. Maziade). The results have been published previously in Schizophrenia Bulletin. However, to have a greater sample size, we used unpublished standard deviations collected from our laboratory since the publication of the paper in Schizophrenia Bulletin. Only one neuropsychological variable, the Stroop from the D-KEFS battery, was gathered from a paper in the literature [32]. The standard deviations for the clinical variables were obtained from published research: Positive and Negative Syndrome Scale (PANSS) [33] and global assessment of functioning (GAF) [32]. We fixed the same test-retest to 0.80 as a standard for each test. The reliability of 0.80 to 0.90 is considered as the minimum acceptable for internal consistency and 0.70 is the minimum for the test-retest reliability [34].

The second approach (less conservative) was to consider the movement of scores from below to above the 16th percentile on instruments with available clinical normative data. In clinical practice, a score above the 16th percentile is considered as normal performance. As a consequence, changes in status from “deficit” to “normal” performance were considered to be a clinical improvement.

## 4. Results

### 4.1. Neuropsychological Assessment

Difference between baseline and posttest assessment scores was calculated, and RCI are reported in Table 3.

The conservative method revealed that Case A significantly improved on RCFT immediate recall (RCI = 2.68) and RCFT delayed recall (RCI = 1.86). The expected improvement in verbal fluency was observed for both categories (phonemic fluency, RCI = 2.20; categorical fluency, RCI = 2.51). However, the expected improvement in selective attention between baseline and posttest assessments was not observed. Case A's score on the Stroop inhibition test remained unchanged, at the threshold of deficit (percentile = 16).

Finally, the less conservative method revealed that difficulties observed at baseline in problem solving (percentile = 11–16) disappeared at posttest assessment (percentile > 16); however, RCI was not significant.

### 4.2. Clinical Assessment

No significant change was observed in clinical symptoms (as assessed with the PANSS) or in social functioning (assessed with the GAF) (Table 3).

### 4.3. Clinical Change during Cognitive Remediation Therapy

The clinical case formulation (see Table 1 in the supplemental material; see Supplementary Material available online at http://dx.doi.org/10.1155/2015/242364) pointed out severe difficulties in verbal fluency but important strengths with good comprehension and high motivation. Verbal fluency was not practiced with specific exercise during cognitive remediation with CIRCuiTS. At the beginning of therapy, Case A was unable to identify examples where the strategies learned in CIRCuiTS might be useful in his daily life. By midtreatment, it was expected that Case A would be able to identify applications in his daily life independently, but this was not the case. Case A's deficit in verbal fluency could prevent him from verbally generating a list of applications during the therapy session. His therapist therefore asked him to make a list of the areas of his life in which he experienced cognitive difficulties (e.g., work, friends, or medication) as homework for the following session. The task was very difficult for him, but, after several drafts, the final version was exhaustive, with concrete examples that reflected his daily life. In the following session, Case A's therapist asked him to use a CIRCuiTS strategy to organize his list. Case A successfully categorized each of the identified situations (e.g., food, interests, and social situations) and the list was used for the remainder of therapy. By asking participants to verbalize strategies during and after training exercises, the CIRCuiTS program helps participants gain awareness of strategies and generate new strategies if application efforts are not successful. The list was an interactive document open to modification and enhancement; it served to keep track of the objectives set at the beginning of therapy. As mentioned in the clinical case formulation (see Table 1 in the supplemental material), Case A's goals were very concrete and directly linked to his daily life (e.g., read a book). The “list” strategy was used because Case A demonstrated deficits in verbal fluency. This deficit made him a unique and interesting case study; however, the strategy is likely to be applicable and useful for other participants, even in the absence of verbal fluency problems.

Case A's problem in verbal fluency also included slowing down during reading and difficulties to synthesize instructions which led to a decrease in the number of exercises performed during a session. This synthesis problem could be due to Case A's cognitive behavioral style (see Table 1 in the supplemental material). Effectively, the clinical case formulation reported some difficulties such as sensitivity to interference or trouble to stay focus/concentrate for a long time. Thus, the first task of Case A was to learn to concentrate by buying the newspaper, choosing an article, reading it alone, and trying to summarize it by writing, taking his time. The strategy chosen by the patient was to highlight important information in the text to bring out the essential meaning. The first summary of Case A looked like a “copy and paste” of the original text, but it became more and more accurate. At the next session, Case A had to read aloud the summary he had previously prepared at home in order to learn to take breaks during his reading time. Then, Case A had to do it again without looking at his paper. The first attempts were quite laborious with too much detail without understanding the main message of the text. The same homework was repeated until Case A was able to summarize a text briefly and to understand its meaning.

Overall, the main strategies that Case A used in therapy and at home were to highlight a text (e.g., in the newspaper), to buy and use a notebook, to categorize the information (e.g., shopping list), to say the information out loud, the self-repetition, to take break during reading, to check his answer before validating it, and to plan before beginning a task. these strategies allowed him to reach two of the three expected goals mentioned in the formulation case, plus four others not expected: read the newspaper, cook, watch movie, play board game, and remember birthdays and phone numbers.

## 5. Discussion

We hypothesized that Case A's performance would improve in each of the cognitive domains identified as impaired at baseline and targeted by CIRCuiTS, including visual memory, inhibition, and problem solving. The hypothesis was partially validated: Case A improved significantly in visual memory (conservative method) and in problem solving (less conservative method). Since verbalization is a strategy widely used in CIRCuiTS cognitive remediation, improvement in verbal fluency was expected even though it was not directly practiced by specific exercise. This second hypothesis was validated. The effects of cognitive remediation were observed only on the neuropsychological measures in this case study. No significant changes were observed in global functioning or in positive/negative symptoms.

Where visual memory was concerned, Case A's improvement was clear: his scores were normal at posttest assessment. Exercises used to train visual memory in the CIRCuiTS program primarily involved copying and recalling images, as well as practicing memory for faces and places. Case A's therapist taught him several strategies for visual memory, including using a grid for visual cues, focusing on one attribute at a time (e.g., face, body, and name), taking notes, and visual scanning (left/right and up/down). Case A seemed to integrate these strategies by the end of therapy, as demonstrated by his improvement in visual memory at posttest assessment. This observation is consistent with recent literature demonstrating improvement in visual memory after basic visual processing training via computer [35].

We expected an improvement in inhibition processes, but none was observed. Case A's impairment remained near the deficit threshold at posttest assessment. According to Lecardeur et al. [36], improvement is rarely observed in individuals with small to moderate deficits (0.5 to 1.5 standard deviation from the norm), and the usefulness of cognitive remediation in such cases is not certain. In the present case study, baseline score was on the edge between deficit and normality (percentile: 16, one standard deviation from the norm) and the opportunity for improvement was therefore limited.

Case A demonstrated improvement (with less conservative method) in problem solving at posttest assessment. Cognitive remediation included several exercises designed to train problem solving, including “seating plan” and “plan-a-day.” In the first type of exercise, Case A's task was to seat individuals at a table while respecting instructions about who should not be seated together. In the second type of exercise, his task was to organize and schedule a list of activities and to-do items in a diary, respecting the instructions given. During the two exercises, Case A used the following strategies: prioritize and follow the simplest rule; place tasks with a fixed time into the schedule first. He learned to dissect instructions before initiating a task and to write down the steps to solve a problem. These strategies appear to have been effective: Case A's problem solving score improved from impaired to within the normal range at posttest assessment. A similar study [37] demonstrated that, at least in the planning/problem solving domains, patients with impaired performance are likely to benefit from interventions with very specific targets. The authors compared the impact of two types of cognitive remediation: specific problem solving training and basic cognition training. The results demonstrated that only specific training (i.e., “plan-a-day” task) resulted in improved problem solving.

Finally, we expected that the verbalization strategy applied in therapy would result in observable improvements in Case A's verbal fluency. This hypothesis was supported for both semantic fluency and phonemic fluency; improvements in verbal fluency were observed during the remediation sessions and Case A also reported improvements in everyday life. That verbal fluency improved despite lack of direct training demonstrates that cognitive remediation had a nonspecific effect. This finding is corroborated by a meta-analysis [38] where computer-assisted cognitive remediation yielded comparable effects in targeted and nontargeted cognitive domains.

In the current case study, one possible explanation for the observed nonspecific improvement is the type of cognitive remediation used in the CIRCuiTS program; namely, the “drill, practice, and strategy” approach. “Drill and practice” remediation does not focus on strategies (e.g., verbalization). Therefore, if it does not focus on verbal fluency exercises making improvement in verbal fluency is unlikely. In contrast, the “drill, practice, and strategy” approach is likely to have a widespread effect and to generate nonspecific improvements [39]. Vianin et al. [40] demonstrated this effect. They measured brain activity during a verbal fluency task in eight patients with schizophrenia (experimental group) before and after participation in a cognitive remediation program that did not target verbal fluency. They compared the results with those of a control group of individuals who did not receive remediation. Following cognitive remediation, neuroimaging results revealed greater activation of Broca's area during verbal fluency tasks in the experimental group compared to in the control group. The authors hypothesized that the observed brain changes were attributable to verbal mediation techniques such as verbalization. Finally, the current case study corroborates the fact that cognitive remediation therapy benefits more to patients with schizophrenia with low initial memory performances [41]. Effectively, Case A's baseline performance was very low in visual episodic memory and this impairment could allow him to have general benefit of cognitive remediation by obtaining specific and nonspecific improvements.

There are several limitations to the present study. First, the case study presented here did not assess the long-term effect of cognitive remediation. It would be interesting to conduct a follow-up assessment of cognitive performance; such an assessment would permit observation of change in social functioning (ongoing project). Second, the case study design is often considered to be less valid than are group designs, because of threats to internal and external validity. However, many authors have argued that single-case studies play an important role in evidence-based clinical practice of cognitive remediation [42]. Finally, the administration of the same neuropsychological battery at baseline and at posttest assessment may have positively influenced posttest scores. Therefore, the problem solving's improvement observed with the less conservative method must be interpreted with caution because it could be due to the practice effect.

## 6. Conclusions

This case study highlights several important points. First, cognitive processes improved when the participant's training focused on specific targets such as visual memory and problem solving. Second, when cognitive remediation includes learning strategies, cognitive processes such as verbal fluency may improve even though they are not practiced by specific exercises. That is, learning strategies can produce generalized improvements and enhance the positive impact of cognitive remediation. In the light of this case study, cognitive remediation appears to be an interesting avenue, workable, and advantageous for patients with schizophrenia. Therefore, the use of cognitive remediation in clinical practice represents an obvious interest and could lead to positive impact on social functioning.

## Supplementary Material

The supplementary material is about Case A's clinical case formulation which describes his difficulties and his strengths concerning cognitive functions, cognitive behavioral style, no cognitive factors, coping and compensation strategies.

## Figures and Tables

**Table 1:** Neuropsychological and clinical assessments.

Cognitive processes	Tests	Variables
Neuropsychological assessment		
Intelligence	Wechsler Adult Intelligence Scale third edition (WAIS-III) [43]	Global intelligence
Verbal episodic memory	The California verbal learning test-II (CVLT-II) [44]	Immediate recall, delayed recall, and recognition
Visual episodic memory	Rey complex figure test (RCFT) [45]	Immediate recall, delayed recall, and recognition
Sustained attention	Continuous performance test-II (CPT-II) [46]	Hit reaction time block change (change in performance over time) Hit standard error block change (accuracy)
Selective attention Inhibitory processes	CPT-II Inhibition score of the Stroop test from the Delis-Kaplan Executive Function System (D-KEFS) [47]	Omissions, commissions, and detectability
Working memory	Span [48]	Total spatial span forward and backward and total digit span forward and backward
Problem solving	Wisconsin card sorting test-128 cards (WCST: CV4) [49]	Trials to complete the first category and failure to maintain set
Initiation/strategic search	Verbal fluency test (French-Canadian version)	Semantic (i.e., animals) and phonemic (i.e., words starting with the letter *“*f*”*) categories
Planning	Tower of London (TOL^DX^) [50]	Number of problems solved in minimum moves, rule violation, and time violation

Clinical assessment		
Psychiatric symptoms	Positive and Negative Syndrome Scale (PANSS) [51]	This instrument includes 30 items rated on a scale from 1 (absent) to 7 (extreme)
Social and occupational functioning	Global assessment of functioning (GAF) [52]	This instrument measures on a scale from 1 to 100, higher scores reflecting better functioning. It is divided into ranges of 10 points (i.e., 1–10, 11–20, etc., up to 91–100)

**Table 2:** Case A's results on neuropsychological and clinical assessments.

	Baseline		Posttest	
	Score	PR	Score	PR
*Cognitive Tests *				
Intelligence				
Global IQ^a^	88	21	95	37
Verbal episodic memory				
CVLT-II total recall	55	55	50	50
CVLT-II delayed recall	14	84	14	70
CVLT-II recognition	16	70	14	70
Visual episodic memory				
RCFT immediate recall	12	1	23.5	50
RCFT delayed recall	20	1	43	24
RCFT recognition	23	86	19	8
Sustained attention				
CPT-hit reaction time^b^	0.05	97.12	0.02	74.66
CPT-hit standard error^b^	0.03	62.12	0.00	47
Selective attention				
CPT omissions^b^	0	20.8	1	30.41
CPT commissions^b^	11	37.24	14	52.37
CPT detectability *d*′^b^	0.62	56.92	0.52	65.3
Stroop D-KEFS inhibition	66	16	64	16
Working Memory				
Total spatial span	20	84	18	63
Total digit span	16	37	18	50
Executive function/problem solving				
WCST total errors	20	42	17	50
WCST number of categories completed	6	>16	6	>16
WCST trials 1st category	13	11–16	11	**>16** ^*^
WCST failure to maintain set	0	>16	0	>16
WCST learning to learn	2.8	>16	−1.52	>16
Executive function/initiation				
Letter fluency test	6	2	12	25
Category fluency test	12	3	21	44
Executive function/planning				
Total number of problems solved with minimal movements	4	40	1	9
Total time violations	0	66	0	66
Total rules violations	0	55	0	55
*Clinical Tests *				
GAF	48	n/a	48	n/a
PANSS	85	n/a	74	n/a
Positive Symptoms Scale	14	n/a	14	n/a
Negative Symptoms Scale	23	n/a	21	n/a
General Psychopathological Scale	48	n/a	39	n/a

^a^Global IQ was reported in standardized score.

^b^The PR of CPT scale is reversed. Higher PR indicates more severe impairment.

Percentile rank:PR. The scores correspond to the raw score obtained for each variable; California verbal learning test-II: CVLT-II; Rey complex figure test: RCFT; global IQ as measured with the WAIS-III; continuous performance test-II: CPT-II; Wisconsin card sorting test-128 cards: WCST; global assessment of functioning: GAF; Positive and Negative Syndrome Scale: PANSS; not applicable (n/a).

^*^Deficit at baseline improved to normal at posttest scores.

**Table 3:** Reliable Change Index (RCI) for the neuropsychological variables.

	RCI
*Cognitive Tests *	
Intelligence	
Global IQ	0.919
Verbal episodic memory	
CVLT-II total recall	−0.799
CVLT-II delayed recall	0.000
CVLT-II recognition	−0.930
Visual episodic memory	
RCFT Immediate Recall	**2.682** ^**^
RCFT delayed recall	**1.863** ^*^
RCFT recognition	−**3.194** ^**^
Sustained attention	
CPT-hit reaction time	−1.581
CPT-hit standard error	−0.565
Selective attention	
CPT omissions	0.166
CPT commissions	0.569
CPT detectability *d*′	−0.386
Stroop D-KEFS inhibition	−0.167
Working Memory	
Total spatial span	−1.068
Total digit span	0.811
Executive function/problem-solving	
WCST total errors	−0.188
WCST number of categories completed	0.000
WCST trials 1st category	−0.083
WCST failure to maintain set	0.000
WCST learning to learn	−1.016
Executive function/initiation	
Letter fluency test	**2.196** ^**^
Category fluency test	**2.514** ^**^
Executive function/planning	
Total number of problems solved with minimal movements	−**1.890** ^*^
Total time violations	0.000
Total rules violations	0.000
*Clinical Tests *	
GAF	0.000
PANSS	−1.214
Positive Symptoms Scale	0.000
Negative Symptoms Scale	−0.497
General Psychopathological Scale	−0.180

Abbreviations: RCI: Reliable Change Index; GAF: Global Assessment of Functioning; PANSS: Positive and Negative Syndrome Scale.

^*^Significant with unilateral criteria (cut off = 1.64); ^**^Significant with bilateral criteria (cut-off = 1.96).
