# Supplementary material for: Top-Down Computerized Cognitive Remediation in Schizophrenia: A Case Study of an Individual with Impairment in Verbal Fluency
Source: Case Rep Psychiatry. 2015 Apr 9;2015:242364. doi: 10.1155/2015/242364 (PMC4408634; doi:10.1155/2015/242364)
Supplement: Supplementary file 1 — The supplementary material is about Case A's clinical case formulation which describes his difficulties and his strengths concerning cognitive functions, cognitive behavioral style, no cognitive factors, coping and compensation strategies. [file 242364.f1.docx]

**Table 1: Clinical case formulation (Wykes & Reeder, 2005, p.226)**

| Patient: Case A | Age (in years and months): 33 years and 7 months |
| --- | --- |
| Education: He had completed 13 years of education and had obtained a professional certificate in institutional plumbing. | |
| Current and previous employment: he has worked as a plumber until 2008, and has been unemployed since 2008. | |
| Current and previous interests: he loved cooking and reading. Now, he likes watching TV. | |
| **Cognitive Functions** | |
| Current estimated full-scale IQ: 88 | |
| Current estimated verbal IQ: 88 | |
| Current estimated performance IQ: 91 | |
| *Difficulties:* | *Strengths:* |
| 1. Information processing speed | 1. Visual working memory |
| 2. Visual episodic memory/Organisation | 2. Verbal episodic memory |
| 3. Verbal fluency |  |
| 4. Inhibition |  |
| 5. Perseveration |  |
| 6. Verbal comprehension |  |
| **Cognitive behavioral style** | |
| *Difficulties:* | *Strengths:* |
| 1. Sensitivity to interference | 1. Good comprehension |
| 2. Word finding problem | 2. self-repetition, verbalization |
| 3. Attentionnal decline at the end of exercise (with high attentional coast) |  |
| **No cognitive factors** | |
| *Difficulties:* | *Strengths:* |
| 1. Anxiety | 1. High motivation |
|  | 2. Watchfull and attentive |
|  | 3. Find strategies himself |
| **Coping and use of compensatory strategies** | |
| *Problem behavioral coping:* | *Effective coping and strategies:* |
| 1. | 1. Verbalization, self-repetition |
| 2. | 2. Highlight/underline relevant information |
| 3. | 3. Use of his own strategies |
| **Problems** | **Goals** |
| 1. Lost of common thread | 1. Read the newspaper |
| 2. To have to be concentrated a long time, to retain information during a long time | 2. Watch a movie without the use of the “pause” or “rewind” buttons |
| 3. | 3. Read a book |
